# Effect of nitrous oxide on pain due to rocuronium injection: A randomised, double-blind, controlled clinical trial

**DOI:** 10.4103/0019-5049.63660

**Published:** 2010

**Authors:** Sonal Sharma, Deepak Sharma, Ashish Jain, Anjali Jain

**Affiliations:** Department of Anesthesiology, SDM Hospital, Jaipur, Rajasthan, India; 1 Department of Anesthesiology and Pain Medicine, University of Washington, Seattle, WA, USA; 2 Department of Preventive and Social Medicine, S.N. Medical College, Agra, India

**Keywords:** Nitrous oxide, pain, rocuronium

## Abstract

A prospective, randomised, double-blind, placebo-controlled study was carried out to determine the effect of nitrous oxide (N_2_O) on the frequency and severity of pain and withdrawal reactions after injection of rocuronium. Eighty ASA physical status I and II patients undergoing general anaesthesia for elective surgery were enrolled. The patients were randomised to receive 100% oxygen (O_2_), or 50% N_2_O in O_2_ for 3 minutes followed by a subparalysing dose of rocuronium 0.06 mg/kg. After induction of anaesthesia with thiopentone 5 mg/kg, an intubating dose of rocuronium 0.6 mg/kg was given. The patients were observed after injection of rocuronium 0.06 mg/kg, and asked to rate pain in the arm on a 4-point (0–3) verbal rating scale (none, mild, moderate or severe). After the intubating dose of rocuronium, withdrawal reactions were recorded. Thirty-six patients (90%) in the group N_2_O and 15 patients (37.5%) in the group O_2_ reported no pain (*P* < 0.001). The pain was mild in 1 (2.5%) and 9 (22.5%) patients in N_2_O and O_2_ groups, respectively (*P* = 0.006). Moderate pain occurred in 2 (5%) patients in group N_2_O and 15 (37.5%) patients in group O_2_ (*P* = 0.001). Severe pain was reported by one patient in each group (*P* = 0.47). Withdrawal response after an intubating dose of rocuronium was observed in 6 (15%) and 18 (45%) patients in the N_2_O and O_2_ groups, respectively (*P* < 0.05). Inhalation of 50% N_2_O in O_2_ reduces the incidence and severity of pain and the withdrawal reactions associated with rocuronium injection.

## INTRODUCTION

Pain during induction of anaesthesia is distressing and it increases the stress and anxiety among patients just before surgery. Rocuronium is a widely used steroidal neuromuscular blocking agent with an intermediate duration and a rapid onset of action. The major disadvantage of rocuronium is the pain associated with its injection, the cause of which is not clear. With a subparalysing dose, 50–100% of patients report discomfort.[1‐5] Even after induction of anaesthesia with propofol or thiopental, rocuronium causes hand or limb withdrawal in 85% of patients, suggesting the presence of intense nociception, even during anaesthesia.[4] A number of treatment modalities have been studied in an attempt to reduce the pain on injection of rocuronium, albeit with variable success rates. These include pretreatment with lidocaine, esmolol, ketamine, opioids, dexmedetomedine and injection of neutralised rocuronium.[3,4]

N_2_O is an analgesic gas that has been used for more than 100 years. It has been used effectively to reduce the pain associated with propofol injection but its effect on reducing pain on rocuronium injection has never been studied. We hypothesised that inhalation of 50% N_2_O reduces the incidence and severity of pain and the withdrawal reactions after injection of rocuronium. Hence, the primary aim of this prospective, randomised, double-blind, controlled trial was to determine the effect of N_2_O on the incidence and severity of pain during injection of a subparalysing dose (0.06 mg/kg) of rocuronium. Our secondary aim was to determine its effect on incidence of withdrawal reactions after administration of an intubating dose (0.6 mg/kg) of rocuronium after induction of anaesthesia.

## METHODS

After obtaining approval from the institutional ethics committee and written informed consent, 80 unpremedicated ASA physical status I or II patients, aged 18–55 years, undergoing general anaesthesia for elective surgery were enrolled. Patients with chronic pain, anticipated difficult airway, pregnancy, contraindication to N_2_O, and patients receiving analgesics or sedatives were excluded from the study. Patients were informed that they would be receiving a drug at the start of their anaesthesia that may make their arm "sting." They were told that they would be asked to score their pain on a 4-point scale (0-3), if any, after the drug had been given. They were, however, informed that they would be blinded to their group assignment.

A 20-G intravenous cannula was placed preoperatively in the largest vein on the dorsum of the hand and an intravenous infusion of sodium chloride 0.9% was started. On arrival at the operating room, all the patients were monitored with an electrocardiograph, pulse oximeter and an automatic noninvasive arterial pressure monitor. The patients were randomly assigned to one of the two groups by drawing of lots (i.e. drawing blindly from the box that contained exactly 80 coded lots; 40 in each group). They received either inhalation of 50% N_2_O in oxygen (O_2_) for 3 minutes (Group N_2_O) or inhalation of 100% O_2_ (control) for 3 minutes. During this time, a screen was placed in front of the flow meters in such a fashion that the investigator collecting the data (pain scoring and observation of withdrawal response) was blinded to the group assigned. Once the patient had inhaled 50% N_2_O-O_2_ mixture or pure O_2_ for 3 minutes, a subparalysing dose of rocuronium 0.06 mg/kg, diluted with saline to an injection volume of 5 ml was injected over 10 seconds. The patients were observed and asked immediately if they had pain in the arm, and their response was assessed according to the verbal rating scale[4] [Table 1]. Anaesthesia was then induced with thiopentone 5 mg/kg. When the eyelash reflex was abolished, an intubating dose of rocuronium 0.6 mg/kg was injected over 10 seconds and withdrawal movements, if any, were recorded. Any adverse effects were also noted. Thereafter, all the patients received fentanyl 2 *µ*g/kg before intubation. Further anaesthesia was continued using drugs and techniques at the discretion of the attending anaesthesiologist.

**Table 1:** Assessment of pain during injection of subparalysing dose of rocuronium[5]

Degree of pain	Response
None (0)	Negative response to questioning
Mild (1)	Pain reported in response to questioning only, without any behavioural signs
Moderate (2)	Pain reported in response to questioning and accompanied by a behavioural sign, or pain reported without questioning
Severe (3)	Strong vocal response or response accompanied by facial grimacing, arm withdrawal or tears

To detect a 50% reduction at a significant level of 5% and a probability of 80%, this study required at least 40 patients per group, estimating the frequency of 80% of patients who experience pain or withdrawal movement on injection of rocuronium. Data are expressed as mean ± SD or numbers (percentages). Statistical analyses were performed using SPSS Windows based version 15.0 (Statistical Package for Social Sciences, SPSS Inc., Chicago, IL, USA). Patient characteristics were analysed using one-way analysis of variance. Chi-square test was used for other statistical analysis (gender and injection related pain or movement). A *P* value of less than 0.05 was considered significant.

## RESULTS

**Figure 1:**
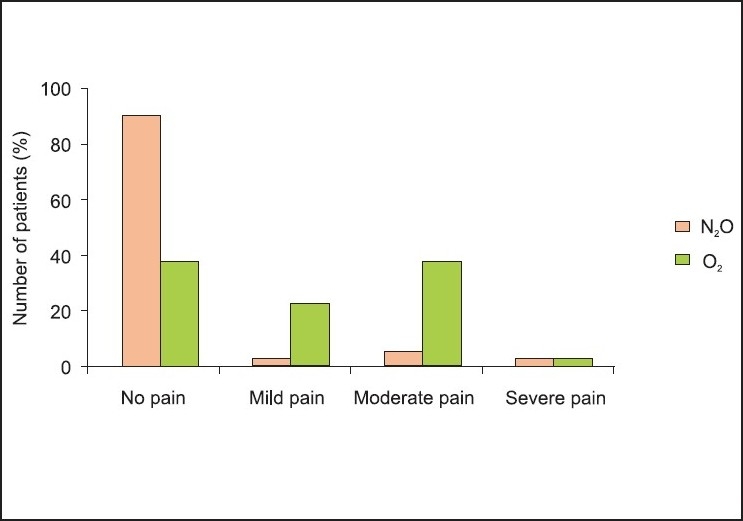
Intensity of pain during injection of subparalysing dose of rocuronium

All the 80 patients completed the study. Demographic data were comparable among the two groups [Table 2]. Figure 1 shows the intensity of pain caused by the subparalysing dose of rocuronium after the inhalation of 100% O_2_ or 50% N_2_O in O_2_. The overall incidence of pain was 62.5% in the O_2_ group and 10% in the N_2_O group (*P* < 0.001). Although the incidence of severe pain was similar among the two groups (*P* = 0.47), 15 (37.5%) patients in the O_2_ group had moderate pain and 9 (22.5%) had mild pain. The inhalation of 50% N_2_O in O_2_ significantly reduced the pain and discomfort reported by patients. One (2.5%) out of 40 subjects in the N_2_O group had mild pain (*P* = 0.006) and two (5 %) had moderate pain (*P* = 0.001 vs. control) [Figure 1].

**Table 2:** Demographic data

	Groups		*P* value	
	N_2_O (*n* = 40)	O_2_ (*n* = 40)
Age (years)	33.9 ± 10.4	33.8 ± 11.0	0.984
Weight (kg)	57.8 ± 10.7	57.6 ± 12.2	0.923
Gender (M/F)	12/28	15/25	0.478

Values are expressed as mean ± SD or numbers

The intubating dose of rocuronium caused fewer withdrawal reactions in the N_2_O group than in the O_2_ group (*P* < 0.01) [Table 3]. None of the patients in either group had oxygen desaturation during the induction of anaesthesia. No other complications attributable to the drugs were noted.

**Table 3:** Withdrawal reactions to intravenous administration of an intubating dose of rocuronium

Limb withdrawal	Groups		*P* value	
	N_2_O (*n* = 40)	O_2_ (*n* = 40)
Present	6 (15)	18 (45)	0.003
Absent	34 (85)	22 (55)	

Values are expressed as numbers (percentages)

## DISCUSSION

Pain associated with administration of rocuronium is common and distressing for patients[1‐5] and has restricted the usage of rocuronium for the prevention of succinylcholine-induced fasciculation and myalgia, where it is reported to have superior effectiveness as compared with other neuromuscular relaxants.[6] The 62.5% incidence of pain associated with rocuronium injection seen in the placebo group in this series is similar to that observed by Yavascaoglu *et al*.[4] although they used smaller dose of rocuronium (0.05 mg/kg) than us. Mencke *et al*.[5] reported 33% incidence of pain using an even smaller dose of rocuronium (0.03 mg/kg in 5 ml saline).

The mechanism of rocuronium-induced pain remains unclear although various theories have been proposed to explain the aetiology of this pain. These include direct activation of C-nociceptors by the osmolality or pH of the solution, or activation by the release of endogenous mediators such as histamine, kinin and other substances mediating inflammation.[1,2,7,8] Although the low pH of rocuronium may be a possible cause, injection of acidic solutions is generally associated not only with pain but also with perivenous oedema and thrombophlebitis, which are rarely seen after rocuronium injection. Local release of mediators is suspected as the likely mechanism due to short lasting nature of the pain with rocuronium. But in the absence of associated erythema, histamine may be an unlikely mediator. However, kininogen cascade similar to that associated with propofol pain may be responsible. Numerous techniques with variable success rates have been used to reduce the incidence and intensity of this pain. These include diluting rocuronium, administering it as an infusion rather than as a bolus injection and the use of other medications such as various combinations of lidocaine with rocuronium, the use of antiemetics, antihistaminics, short acting opioids (fentanyl, alfentanil and remifentanil), sodium bicarbonate, ketamine, dexmedetomidine and esmolol.[4,9‐15] The most popular methods involve lidocaine or sodium bicarbonate pretreatment or mixing these drugs with rocuronium.[4,9,10] However, even with the use of these drugs, the incidence of pain has been reported to be 20–28% with lidocaine and 10–14% with sodium bicarbonate.[4,9,10]

Rocuronium can also cause pain even after induction of anaesthesia resulting in withdrawal movements. These withdrawal movements may negatively affect the patient outcome.[12] Lui *et al*. reported a child who developed pulmonary aspiration secondary to gastric regurgitation caused by spontaneous movements after the injection of rocuronium.[16] Pain, emotional stress and stimulation during induction of anaesthesia may cause bronchospasm or myocardial ischaemia and the withdrawal movements may dislodge the venous catheter or cause injury during induction.[17]

The present study was conducted to evaluate if the inhalation of 50% N_2_O in O_2_ could alleviate pain on rocuronium injection and reduce the withdrawal response to injection because its analgesic efficacy is well known and it has previously been used successfully to alleviate pain associated with injection of propofol. Sinha *et al*. have reported the use of N_2_O in reducing pain on propofol injection in adult patients.[18] They compared the efficacy of pretreatment with N_2_O (with or without premixed lignocaine in propofol) for the prevention of propofol-induced pain and found a significant decrease in the incidence of pain from 36.7% (in patients who received 50% O_2_ in air with lignocaine mixed in propofol) to 3.3% with use of 50% N_2_O in O_2_ along with the above mentioned regime. While Harmon *et al*. reported inhalation of N_2_O in oxygen O_2_ to decrease pain on propofol injection with similar efficacy to the admixture of 20 mg lidocaine in propofol,[19] Niazi *et al*. found that combination of intravenous lidocaine and N_2_O in O_2_ inhalation pretreatment was more effective than either treatment alone in decreasing pain on propofol injection.[20]

In the present study we found that the incidence of pain on injection of rocuronium was significantly reduced if the patients inhaled 50% N_2_O in O_2_ (*P* < 0.001). These results are comparable to the results obtained with the use of other agents by different investigators. Yavascaoglu *et al*. found that esmolol and lidocaine reduced the frequency of pain associated with rocuronium injection, the incidence of no pain being 77.5% in the esmolol group and 80% in the lidocaine group (both groups vs. placebo, *P* < 0.001).[4] Chiarella *et al*. compared the addition of fentanyl 100 *µ*g, lidocaine 2% and sodium bicarbonate 8.4% to 10 mg of rocuronium and found that addition of fentanyl or lidocaine reduced the complaint of pain reported by patients by 1.9 times (*P* < 0.049) and 3.6 times (*P* < 0.0001), respectively.[9] Sodium bicarbonate reduced the complaint of pain by 18.4 times (*P* < 0.0001).[8] These results were similar to that found by Han DW *et al*. in their study using sodium bicarbonate.[10] The frequency of withdrawal response with rocuronium after induction of anaesthesia in our control group was 45%, which was decreased by inhalation of N_2_O to 15% that is comparable to the results obtained by Yavascaoglu *et al*. in their study using lidocaine (incidence of withdrawal movements in lidocaine group 17.5%).[4]

N_2_O is a centrally acting sedative and an analgesic agent, commonly used in anaesthetic practice. It is widely available and easy to administer. It is inexpensive and relatively free from side effects and has been used for many years to provide analgesia for various day care procedures.[21] It has been used effectively in labour analgesia and interventional radiological procedures.[22,23] While other medications can be used to reduce pain caused due to rocuronium injection, they all have their own side effects and disadvantages. The obvious limitation and caution to the use of N_2_O in limiting pain on rocuronium injection is in patients with anticipated difficult airway who need preoxygenation with 100% O_2_. Moreover, N_2_O has its own well-known contraindications. However, since N_2_O is known to alleviate pain on propofol injection and we have shown that it reduces the pain associated with rocuronium injection, N_2_O may be particularly useful in cases where a combination of propofol and rocuronium is used for induction.

In conclusion, inhalation of 50% N_2_O is a simple and effective method to reduce pain and withdrawal response on rocuronium injection. Further studies are required to compare its efficacy with other drugs and methods in reducing pain.
